# Point prevalence survey to assess antibiotic prescribing pattern among hospitalized patients in a county referral hospital in Kenya

**DOI:** 10.3389/frabi.2022.993271

**Published:** 2022-10-26

**Authors:** Moses Kamita, Michael Maina, Racheal Kimani, Robert Mwangi, Dominic Mureithi, Cynthia Nduta, Jesse Gitaka

**Affiliations:** ^1^ Directorate of Grant and Development, Mount Kenya University, Thika, Kenya; ^2^ Department of Pharmacy, Kiambu Level 5 Hospital, Kiambu, Kenya; ^3^ Department of Animal Sciences, Chuka University, Chuka, Kenya

**Keywords:** point prevalence, antibiotic use, Kenya, antibiotic stewardship (ABS), referral hospital

## Abstract

Antibiotic resistance causes higher morbidity and mortality and higher healthcare costs. One of the factors influencing the emergence of antibiotic resistance is the inappropriate use of antibiotics. Clinical practitioners’ incorrect prescription patterns and a disregard for antibiotic usage recommendations are the leading causes of this resistance. This study examined the antibiotic prescription patterns among hospitalized patients at the Kiambu Level 5 hospital (KL5) to find potential for hospital quality improvement. This study was conducted in July 2021, and all patients hospitalized on the study day were included. The information was extracted from patient medical records using a World Health Organization Point Prevalence Survey (PPS) instrument. Anonymized data was gathered, entered, and then SPSS version 26 was used for analysis. Among the 308 surveyed patients, 191 (62%) received antibiotic medication, and 60.1% of the total were female. The pediatric ward, which had an antibiotic prescription rate of 94.1%, had the highest rate of antibiotic usage, followed by the medical ward (69.2%) and gynecological ward (65.6%). Over 40% of antibiotic prescriptions had a prophylactic medical indication. Penicillin G was the most prescribed antibiotic for community-acquired infections (32.2%), followed by 3rd generation cephalosporins (27.6%) and aminoglycosides (17.2%). Based on the AWaRe classification, 57% of the prescribed antibiotics were in the Access class while 42% were in the Watch class. Incomplete site of indication, lack of a method of administration, and length of administration are some of the conformities that were missing in the medical records. This study shows that antibiotic prescription rates are high, particularly for young patients, and there is a higher risk of antibiotic misuse. The data makes a compelling justification for using antibiotic stewardship practices in Kenyan hospitals.

## Introduction

Antibiotics have been at the forefront of the fight against infection-related mortality and morbidity (Fourie et al., 2018). However, antibiotics must be used in a timely and appropriate manner to prevent antimicrobial resistance (Mulwa, 2014; Saleem et al., 2019; Afriyie et al., 2020; Saleem et al., 2020). Antibiotic misuse and abuse, particularly of broad-spectrum antibiotics, leads to the development of resistance (Saleem et al., 2019; Afriyie et al., 2020; Saleem et al., 2020). Antibiotic resistance has become a major concern, with a devastating trend associated with poor clinical outcomes and increased treatment costs (Mulwa, 2014; Ginting et al., 2019).

Antimicrobial stewardship (AST) has been incorporated into national action plans (NAPs) of low and middle-income countries (LMICs) to combat antimicrobial resistance (Seale et al., 2017; Saleem et al., 2019; Antonioli et al., 2020). Point prevalence surveys (PPS) serve as the backbone of the feedback loop for healthcare providers and policymakers in responding to antibiotic resistance policies (Elhajji et al., 2018; Kurdi et al., 2021). National standard treatment guidelines (NSTGs) are being developed in LMICs to raise awareness through the Drugs and Therapeutics Committees (DTCs). Antibiotic use in China (Xie et al., 2015) and Egypt (Umeokonkwo et al., 2019) has been reported to be up to 59% among in-patients. Most African countries report that most prescriptions are done empirically (Talaat et al., 2014; Nsofor et al., 2016; Labi et al., 2018; Umeokonkwo et al., 2019). There is a rising concern about high rates of antibiotic resistance across Africa, Asia, and Europe (Fourie et al., 2018), with accumulating reports indicating increased prescription of second and third-line antibiotics (Labi et al., 2018; Kurdi et al., 2021).

In Kenya, studies have found a high prevalence of inpatient antibiotic use, with some hospitals recording a prevalence close to 80% in inpatient departments (Mulwa, 2014). To date, only a handful of hospitals have had point prevalence studies on antibiotic use conducted here in Kenya (Okoth et al., 2018; Momanyi et al., 2019; Omulo et al., 2020; Maina et al., 2021). There is, therefore, a need to extend these studies to other hospitals to get a more representative figure on the prevalence of antibiotic use in Kenya. This study aimed to determine the variation in the quality and quantity of antibiotics prescribed to patients admitted to Kiambu Level 5 (KL5) hospital. The goal was to identify quality improvement targets and help the hospital design interventions for prudent antibiotic usage.

## Methodology

### Study site

The study was carried out at KL5 hospital, one of two major referral public hospitals in Kiambu County in Kenya. The hospital has an annual admission of up to 18,525 patients with a bed capacity of 426 beds.

### Study design and target population

The study employed a point prevalence cross-sectional design to determine the variability in the use of antibiotics in all the wards in KL5 as well as to characterize the types of antibiotics used. The study targeted all the patients in the different wards, ranging from children to adult wards. Universal sampling was used, and this included all the patients admitted to the wards. Information about patient prescriptions and indications was collected at 8.00 am on the survey day since this timing was deemed appropriate. The timing did not interrupt the ward rounds and excluded patients admitted later in the day.

### Data collection

Data collection took place between July 7^th^ and July 16^th^ 2021, and was collected using standardized forms from the Global PPS protocol with a few modifications (World Health Organization, 2018; Zumaya-Estrada et al., 2021). Each ward was allocated its day for data collection to ensure uniform sampling time was applied in all wards. A set of forms was used to collect the different data types. These forms were the hospital form, ward form, and patient form. The hospital form gathers information about the hospital, including the type of the hospital, the number of wards and the beds contained, and bed occupancy. The ward form collected data on the wards, including the dates the survey was conducted, the number of patients admitted to the ward, the type of the ward, and the capacity of the ward. The other form was the patient form which was used to gather data on each patient including their demographic characteristics, antibiotics administered if any, route, dose, duration, and indication among other variables. The indication form collected data on the indication for antibiotic use. The variables included in the form were the nature of the infection, the site of diagnosis, the date the treatment was started, and laboratory tests that were conducted. The antibiotic form collected data on the name of the antibiotics prescribed, dose, route, frequency, duration of use, diagnosis, and indications. Clinical pharmacists stationed in KL5 hospital were engaged to help with data collection. The data source was patient medical files which were collected in the morning and taken to a centralized place within the ward for data abstraction.

### Antibiotic usage definitions

Indications for antibiotics were divided into either therapeutic or prevention, while infections were classified as either community-acquired (symptoms present on admission or less than 48 hours after admission) or hospital-acquired infections (symptoms occurring more than 48 hours after admission). The preventive antibiotics were also classified as either medical or surgical. Antibiotic prescription data were obtained from the treatment sheets.

### Statistical analysis

After cleaning the data in Microsoft Excel, it was exported to the Statistical Package for the Social Sciences (SPSS) version 26 for analysis. To summarize findings, descriptive statistics were used, while categorical data was summarized as counts and percentages. The results are presented in tables and figures. Antibiotics were distributed into the AWaRe classes as per the WHO AWaRe classification, 2021 (WHO, 2021).

### Ethical consideration

Ethical approval to undertake the study was sought from Mount Kenya University ethical committee (Ref No. MKU/ERC/889) and a license to conduct the study was obtained from the National Commission of Science, Technology, and Innovation (NACOSTI) [NACOSTI/P/21/10948]. Authorization to conduct the study was also sought from Health Research Development in Kiambu County and the Kiambu Level 5 hospital administration.

## Results

The wards had a total of 317 beds. Only one bed in the intensive care unit was occupied. The pediatric ward had the highest bed occupancy (94.4%, n = 34), followed by the gynecology ward (94.1%, n = 32). On the survey days, 308 patients were admitted to the hospital, resulting in a 97.2% occupancy rate (**Table 1**).

**Table 1:** Bed capacity and occupancy in Kiambu hospital.

Ward	Number of beds	Number of patients admitted to the ward	Bed occupancy per ward (%)
Intensive Care Unit	1	1	100
Pediatrics	36	34	94.4
Gynecology	34	32	94.1
Male Medical	49	45	91.8
Female Medical	36	31	86.1
Female Surgical	39	33	84.6
Newborn Unit	53	44	83
Post Natal Ward	43	32	74.4
Labor Ward	31	22	71
Male surgical Ward	49	34	69.4
Totals	317	308	97.2

Most of the patients in all the wards were aged between 20 and 64 years old (182, 59.1%), with female being the majority (185, 60.1%). The top-3 wards that had the highest number of patients were surgical wards (78, 25.3%), medical wards (65, 21.1%), and newborn units, accounting for 14.3% (n = 44) of all the patients (**Table 2**).

**Table 2:** Baseline characteristics of participants at Kiambu Level 5 hospital.

Characteristics		n (%)
Age (Years)	<5	80 (26)
	5-12	6 (1.9)
	13-19	12 (3.9)
	20-64	182 (59.1)
	>65	27 (8.8)
Sex	Male	123 (39.9)
	Female	185 (60.1)
Wards	Surgical Ward	78 (25.3)
	Medical Ward	65 (21.1)
	Newborn Unit	44 (14.3)
	Pediatric Ward	34 (11.0)
	Postnatal Ward	32 (10.4)
	Gynecology	32 (10.4)
	Labor Ward	22 (7.1)
	Intensive Care Unit	1 (0.3)

Overall, of the 308 patients admitted to all the wards, 62% (n = 191) of the patients were on one or more antibiotics. The ward that had the highest prevalence of antibiotic use was the pediatric ward at 94.1%, followed by the medical wards, gynecology, surgical, and postnatal wards at 69.2%, 65.6%, 64.1%, and 56.3%, respectively (**Table 3**).

**Table 3:** Prevalence of antibiotic use in Kiambu Level 5 hospital.

Patient	No. of Patients	Patients on Antibiotics	Prevalence
Characteristics	(n=308)	(n=191)	(%)
Intensive Care Unit	1	1	100
Pediatric Ward	34	32	94.1
Medical Ward	65	45	69.2
Gynecology	32	21	65.6
Surgical Ward	78	50	64.1
Postnatal Ward	32	18	56.3
Newborn Unit	44	20	45.5
Labor Ward	22	4	18.2
Total	308	191	62

There were a total of 650 antibiotics that were prescribed to all 308 patients. Therapeutic use of antibiotics was more prevalent than prophylactic use (47.4% vs. 52.6%, respectively). Among those antibiotics that had a defined site, gynecological sites were the most common anatomical site for both medical and surgical prophylactic use at 48.9%, followed by skin, soft tissue, bone, and joints (46.7%), and the gastrointestinal tract (4.4%). Sites indicated for antibiotic treatment were skin, soft tissue, bone, and joints, accounting for 26%, followed by respiratory tract infections at 26% and gynecological sites at 17.2% (**Table 4**).

**Table 4:** Distribution of antibiotic use by anatomical site.

Use of antibiotics					
	Total (n=650)		Prophylaxis (n=308) (47.6%)	Treatment (n=342)(52.6%)	
No defined site		378 (58.2)	263 (85.3)		115 (33.6)
Skin, soft tissue, bone, and joints		80 (29.4)	21 (46.7)		59 (26)
Gynecological		61 (22.4)	22 (48.9)		39 (17.2)
Respiratory tract		59 (21.7)	0 (0)		59 (26)
Neonatal		42 (15.4)	0 (0)		42 (18.5)
Gastro-intestinal tract		17 (6.3)	2 (4.4)		15 (6.6)
Central nervous system		9 (3.3)	0 (0)		9 (4)
Urinary tract		2 (0.7)	0 (0)		2 (0.9)
Cardiovascular system		1 (0.4)	0 (0)		1 (0.4)
Ear, nose, and throat		1 (0.4)	0 (0)		1 (0.4)
Total		272	45		227

**Figure 1:**
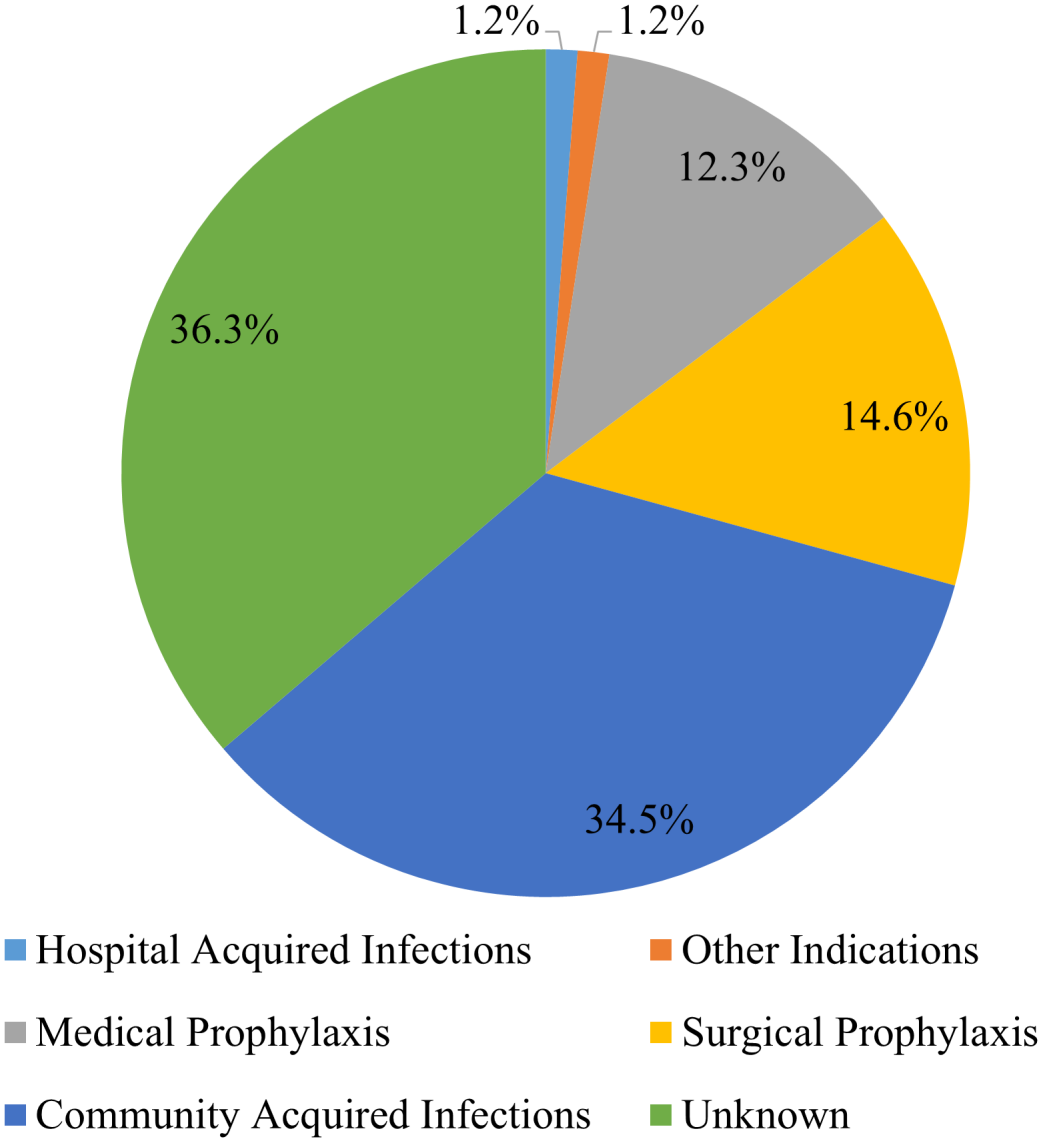
Distribution of patients on antibiotic treatment by indication.

Among the different indications recorded, the most common indication for antibiotic use was community-acquired infections (35%), followed by surgical prophylaxis (15%). There were, however, 36% of the antibiotics whose indications were not reported in the patient files (**Figure 1**).

**Figure 2:**
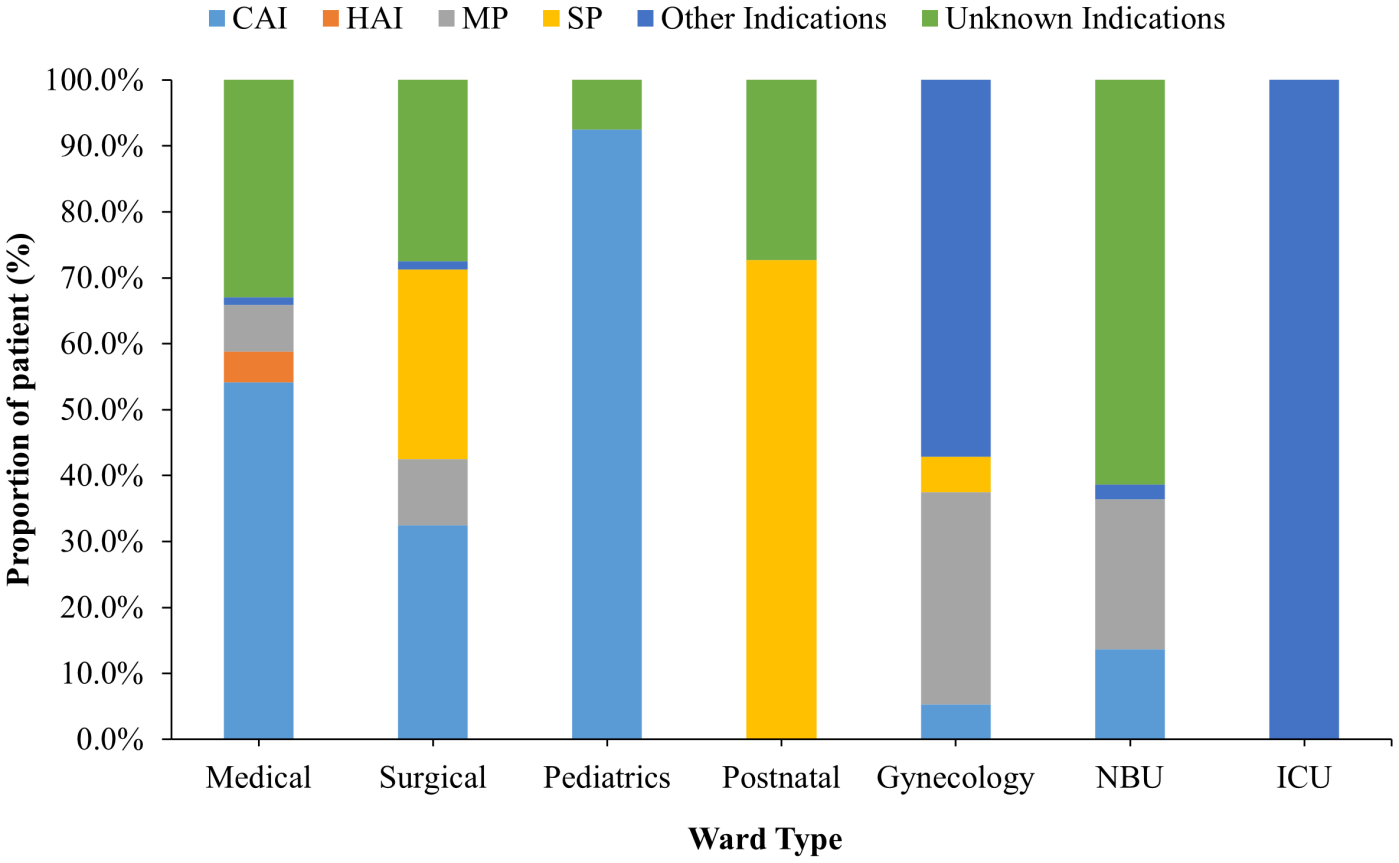
Patient distribution by indication and stratified by hospital wards for those receiving antibiotic treatment. NBU, Newborn Unit; ICU, – Intensive Care Unit.

The proportional frequency of each antibiotic use indication also varied from one ward to another. For instance, whereas community-acquired infections (CAIs) accounted for most of the antibiotic usage in the medical ward (54.1%), most of the antibiotic usage in the postnatal ward (92.5%) was for surgical prophylaxis (SP). However, in the gynecological ward, medical prophylaxis (MP) accounted for 32.1% of all indications (**Figure 2**).

Most antibiotics were prescribed once daily (25.0%), with those prescribed twice daily (19.0%) coming in second. However, more than 26% of the antibiotics lacked an indication of their frequency. Parenteral administration (54.0%) was the most popular route of administration, followed by oral administration (20.8%). Use could last from one day to 14 days. Most of the antibiotics that were in use—roughly 223 (44.6%), had a duration of usage of between one and four days (**Table 5**).

**Table 5:** Frequency, route of administration, and duration of antibiotic use among the patients admitted to Kiambu Level 5 hospital.

Variable	Parameter	n (%)
Frequency	Not Indicated	131 (26.2)
	Once daily	125 (25)
	Twice daily	95 (19)
	Three times a day	60 (12)
	STAT	46 (9.2)
	Four times a day	43 (8.6)
Route	Parenteral	270 (54)
	Not Indicated	126 (25.2)
	Oral	104 (20.8)
Duration of use (days)	1-4	223 (44.6)
	5-9	79 (15.8)
	10-14	9 (1.8)
	>15	28 (5.6)
	Not indicated	161 (32.2)

**Figure 3:**
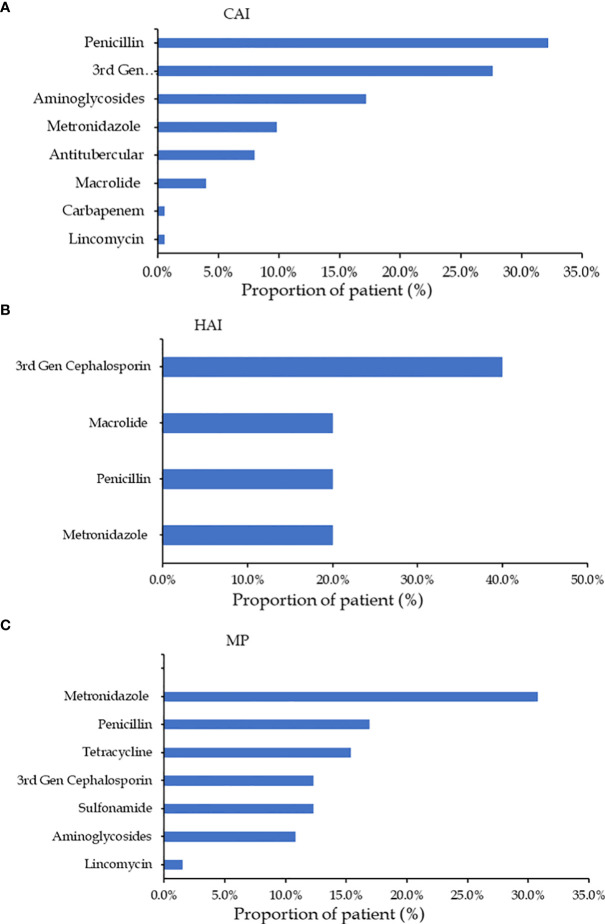
**(A)** Consumption of antibiotics prescribed for community-acquired infections (CAI); **(B)** consumption of antibiotics prescribed for hospital-acquired infections >(HAI) and **(C)** consumption of antibiotics prescribed for medical prophylaxis (MP).

Depending on the type of indication, different antibiotics were employed. For instance, penicillin G was the most frequently given antibiotic (32.2%), followed by 3rd generation cephalosporins (27.6%), and aminoglycosides (17.2%), for community-acquired illnesses (See **Figure 3A**). On the other hand, third-generation cephalosporins (40%) were the most used antibiotics for hospital-acquired infections (**Figure 3B**), while nitroimidazole analogs (30.8%) was the most used medication for medical prophylaxis (**Figure 3C**).

**Figure 4:**
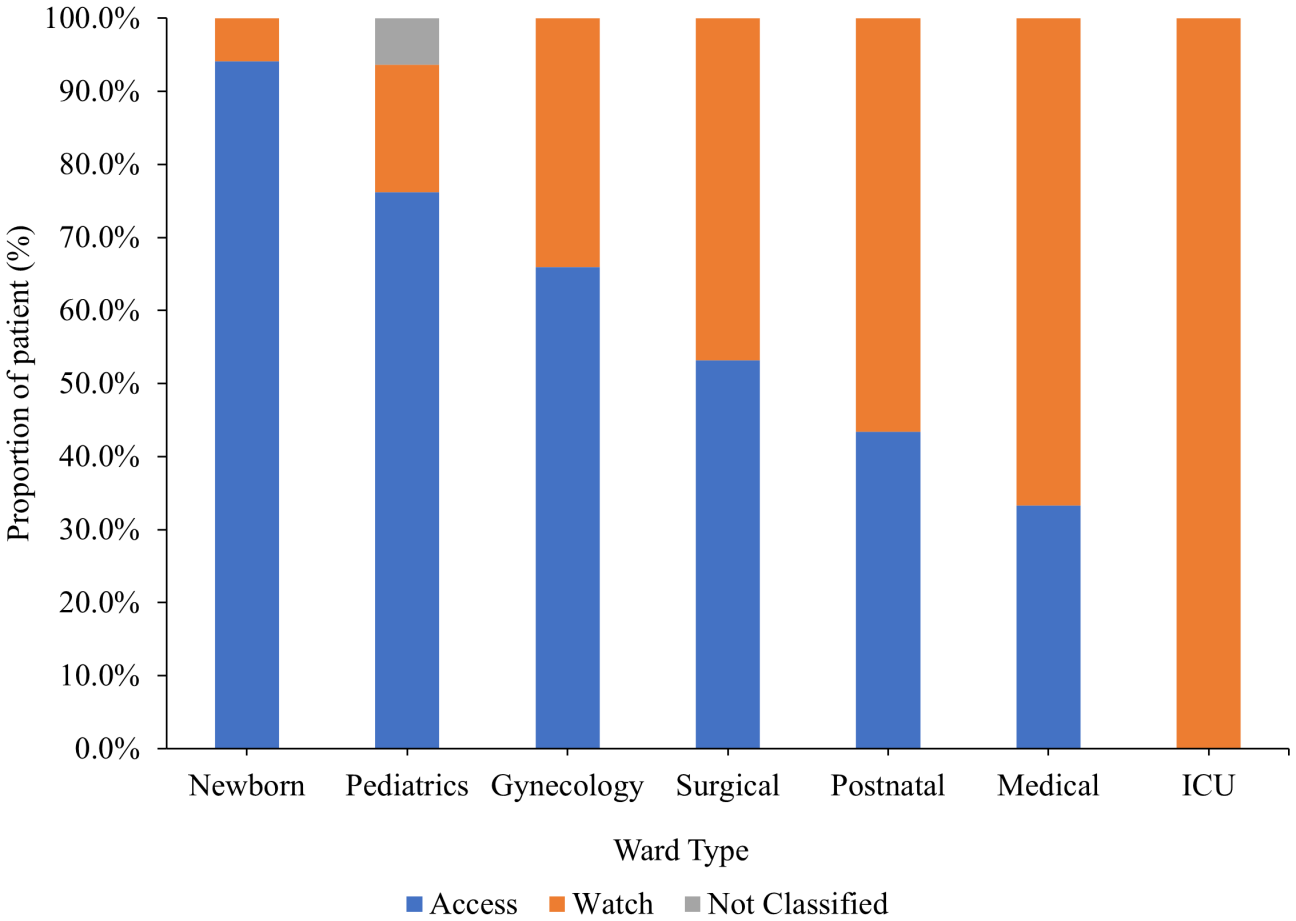
Antibiotics usage by ward type according to the WHO AWaRe classification. ICU, Intensive Care Unit.

Classified by the AWaRe classification, 57% of the antibiotics prescribed were in the Access class while 42% were in the Watch class. Only one percent of the antibiotics were not classified and comprised mainly of antitubercular drugs. Overall, Access class antibiotics were highly used in Newborn unit while Watch class antibiotics were highly used in medical wards (**Figure 4**).

## Discussion

This study discovered a high prevalence of antibiotic prescribing, with 62% of all hospitalized patients receiving antibiotics during the study period. This finding exceeds the 54.7% prevalence reported in a referral hospital in western Kenya (Momanyi et al., 2019) but it is lower than the reported 74% in a study conducted in Makueni, Kenya (Mulwa, 2014). Our study’s prevalence of 62% compares favorably with other similar studies conducted in other parts of the country, ranging from 58.2% to 67.7% (Okoth et al., 2018; Momanyi et al., 2019) as well as other studies in African countries (Talaat et al., 2014; Nsofor et al., 2016; Labi et al., 2019). The prevalence of antibiotic use varies from one hospital to another in Kenya and seems to depend on the capacity of a particular facility and not necessary on their hierarchy level. For instance, while both Kiambu hospital (this study) and Rift Valley Provincial General hospital (Momanyi et al., 2019) are both level 5 hospitals, Rift Valley Provincial General hospital is more equipped than Kiambu Level 5 hospital. This can explain why the prevalence of antibiotic use is lower in Rift Valley Provincial General Hospital than Kiambu Level 5 hospital. Similarly national teaching and referral hospitals have been reported to have much lower antibiotic prevalence (Omulo et al., 2020) than lower level facilities. These results indicate that efforts to ensure judicious use of antibiotics is more of a facility effort than general health practice across health facilities. In comparison to research conducted in developed nations where antibiotic use and standards are strictly adhered to through well-established antibiotic stewardship programs (Versporten et al., 2018), the finding is significantly higher. Due to insufficient laboratory capacity to assist in the detection of specific diseases, broad-spectrum antibiotics are typically prescribed empirically, which accounts for the high prescription rate in this hospital.

When compared to other wards that primarily house adult patients, the pediatric ward’s rate of antibiotic prescriptions was extremely high, at 94.1%, according to our study. This occurrence could be linked to local hospital policies recommending antibiotic treatment for postpartum women and newborns with low birth weight and hypoxia. This finding is in line with research from other African countries, where several studies (Amaha et al., 2018; Apostolakos et al., 2019) reported proportions of patients under the age of five that were higher than 90%. The main causes of antibiotic usage were community-acquired illnesses and surgical prophylaxis (35% and 15%, respectively), which is consistent with findings from other studies (Seni et al., 2020). Overall, the majority (52.6%) of prescriptions were for therapeutic purposes. This result is consistent with research from other African nations (Maina et al., 2017; Anand Paramadhas et al., 2019; Afriyie et al., 2020). Without the aid of a laboratory, the bulk of antibiotic prescriptions is made empirically. Empirical prescribing is mainly caused by difficulties such as a shortage of laboratory technicians, insufficient laboratory supplies, patient cost apprehensions, and patients’ delayed presentation to the hospital after self-medication with antibiotics fails (Dat et al., 2022).

Penicillin G, third-generation cephalosporins, and aminoglycosides were the most frequently prescribed antibiotics among community-acquired infections, accounting for 32.2%, 27.6%, and 17.2% of all prescriptions, respectively. Penicillin G and cephalosporin use have significantly increased globally over the past ten years because of higher health care spending (Amdany and Mcmillan, 2014) and easier access to medications (Maina et al., 2020). Similar studies in poor nations have also found that the use of cephalosporin is preferred over other antibiotics with numerous daily doses in situations where hospital staff is shorthanded because of the frequency of administration and the broad-spectrum nature of the drug (Labi et al., 2018). Metronidazole (30.8%) and penicillin G (32.2%) were the two drugs most frequently used for medical prophylaxis, respectively. The two antibiotics are frequently used for surgical prophylaxis and the treatment of invasive infections (Amdany and Mcmillan, 2014) because they are readily available and reasonably priced in Kenya. WHO has provided a list of antibiotics grouped into three major classes to support antibiotic stewardship. These classes are Access, Watch and Reserve classes. In this study, only 57% of the antibiotics prescribed were in the Access class while 42% were in the Watch class. The use of 42% of the antibiotics in the Watch class is higher than rates reported in Ghana (Amponsah et al., 2021) and Finland (Hsia et al., 2019). However, the use of the Watch class antibiotics was relatively lower than reported in a study conducted in Bangladesh where 64% of the prescribed antibiotics were in the Watch class. Similar to a previous study (Rashid et al., 2022), medical wards had the highest use of antibiotics in the Watch class.

The parenteral route was the most used (54.0%), followed by the oral route (20.8%). Other studies have found that the parenteral route is the most prescribed route of drug administration (Thu et al., 2012). This observation may be explained by the late presentation of critically ill patients to the hospital, the patient’s advanced age, and a contraindication to oral intake. This situation is consistent with the most prescribed antibiotics, penicillin G, and cephalosporins, which are primarily available as parenteral injections. A reduced parenteral route of administration is considered a better antimicrobial stewardship practice and, thus, a good metric for stewardship in a hospital (Amdany and Mcmillan, 2014).

## Conclusion

This study demonstrates the widespread use of antibiotics, particularly in pediatric patients. Most prescriptions are empirical, without the guidance of laboratory microbiological testing. The primary indications for antibiotic use are community-acquired infections and surgical prophylaxis. A significant portion of antibiotic prescriptions was for penicillin G, third-generation cephalosporins, and aminoglycosides. Most medications were in the form of parenteral drug formulations. As demonstrated by this study, there is a risk of inappropriate antibiotic use due to incomplete prescriptions. The study makes a compelling case for implementing antimicrobial stewardship policies that can help reduce empirical treatment and increase the use of more specific and targeted prescriptions of oral antimicrobial formulations. There is a need for a more detailed study to assess the reason behind these prescription practices by the clinicians.

## Data availability statement

The raw data supporting the conclusions of this article will be made available by the authors, without undue reservation.

## Ethics statement

The studies involving human participants were reviewed and approved by Ethics and Review Committee, Mount Kenya University. Written informed consent for participation was not required for this study in accordance with the national legislation and the institutional requirements.

## Author contributions

JG and DM conceptualized the study. JG, MK and RM designed the study. MK, MM, RK, RM, and CN collected data. MK and MM analyzed the data. MK, MM and DM prepared the first draft manuscript. All authors contributed to the article and approved the submitted version.

## Funding

This work was supported by The Kenya National Research Fund (grant number NRF/MKU/2017/007 to JG).

## Conflict of interest

The authors declare that the research was conducted in the absence of any commercial or financial relationships that could be construed as a potential conflict of interest.

## Publisher’s note

All claims expressed in this article are solely those of the authors and do not necessarily represent those of their affiliated organizations, or those of the publisher, the editors and the reviewers. Any product that may be evaluated in this article, or claim that may be made by its manufacturer, is not guaranteed or endorsed by the publisher.
